# Study on Effects of Titanium Surface Microporous Coatings Containing Zinc on Osteoblast Adhesion and Its Antibacterial Activity

**DOI:** 10.1155/2017/2906575

**Published:** 2017-10-02

**Authors:** Quan-ming Zhao, Guang-zhong Li, Hao-ming Zhu, Li Cheng

**Affiliations:** ^1^Department of Orthopedic Surgery, Wuxi People's Hospital, Nanjing Medical University, Wuxi City, Jiangsu Province 214023, China; ^2^State Key Laboratory of Porous Metal Materials, Northwest Institute for Nonferrous Metal Research, Xi'an, Shaanxi Province 710016, China

## Abstract

Metal surface structure/biomedical function integration is the current research focus. In previous studies, we have successfully prepared the microporous coatings containing zinc on the pure titanium surface by MAO. In the study, osteoblasts were seeded on the surface of the microporous coatings containing zinc and the adhesion of osteoblasts were evaluated, and the antibacterial activity of the microporous coatings containing zinc is observed through in vitro bacterial experiments. The result indicates that the adhesion ability of osteoblasts on the surface of microporous coatings containing zinc was very good, and the coatings could obviously inhibit the growth of *Staphylococcus aureus* and had good antibacterial activity. In conclusion, the microporous coatings containing zinc on titanium surface have good osteogenic and antibacterial properties and have good application prospect.

## 1. Introduction

Based on the good biocompatibility, mechanical properties, and corrosion resistance, titanium-based metal has become the first choice for the hard tissue repair and replacement, which has been widely used in clinical practice and achieved good results. But the titanium-based metal is an inert material, and its biological activity is poor after being implanted in vivo, only the mechanical combination with bone tissue can be achieved, and it is difficult to form osseointegration with the bone tissue [1]. It is also prone to result in implant-related infection after implantation [2]. In addition, the titanium-based metal will be corrosive in the body, and metal ions generated during the corrosion not only are harmful to humans but also easily lead to loosing and sinking of the implant, which not only prolongs the implant repair cycle but also affects the success rate of implantation, so it cannot fully meet the clinical needs [3]. In order to make the titanium metal to retain its excellent mechanical properties and also be equipped with good biological activity, which can induce cell adhesion and proliferation after implantation, with good antibacterial properties, the most effective way is to perform surface modification on titanium metal [4].

Through the surface modification, preparing bioactive coatings on the surface of titanium-based metal cannot only maintain the excellent mechanical properties of titanium-based metal but also give biological activity to titanium-based metal, which is a hot spot in recent years. Microarc oxidation (MAO) is an emerging material surface modification developed in recent years. Through this technology, porous, rough ceramic film can be produced in situ of the metal surface [5]. The ceramic film increases the wear resistance, corrosion resistance, and fatigue resistance of the material, greatly improving the surface properties of the material, and because the ceramic film grows in situ of the substrate, and is closely combined with the substrate, it does not easily fall off. It is more attractive that the technology can introduce bioactive elements or antibacterial elements into the coating, greatly improving the material's biological activity, so it is widely used in surface modification of biological materials. Zinc is an important trace element in the human body. It is involved in the structure and function of more than 300 kinds of enzymes in the body, which affects the biological behavior of cells. At the same time, zinc can activate alkaline phosphatase and promote the deposition of callus calcium salt and contribute to the formation and calcification of the bone, thereby promoting fracture healing, and more importantly, zinc has been confirmed to have a good antibacterial effect [6].

In previous studies, we have successfully prepared the microporous coatings containing zinc on the pure titanium surface by MAO. The results showed that the coating material has good surface properties, but further research on the biological behavior and antibacterial properties of the coating material is needed. Therefore, this study will investigate the adhesion of osteoblasts on the surface of microporous coatings containing zinc and the antibacterial activity of the coating material on the basis of previous studies and lay a theoretical foundation for the clinical application of the coating material.

## 2. Materials and Methods

### 2.1. Preparation of Microporous Coatings Containing Zinc on the Pure Titanium Surface

The preparation of the sample has been described in detail in the previous articles [7].

### 2.2. Evaluation of Adhesion and Extensibility of Osteoblasts on the Surface of Microporous Coatings Containing Zinc

The osteoblasts were inoculated into a 25 cm^2^ cell culture flask at 2.5 × 104/cm^2^, and 5 ml of *α*-MEM medium was added to the culture flask. The cells were incubated in a 5% carbon dioxide incubator at 37°C. After cell growth and fusion, the passage was prepared and the old medium was discarded, and after PBS rinsing, the appropriate amount of 0.25% trypsin-0.02% EDTA was added for digestion. The passage began after the medium neutralized the trypsin.

When the third generation of osteoblasts was cultured to 80% of cell fusion, the appropriate amount of 0.25% trypsin-0.02% EDTA was added for digestion, and the trypsin was neutralized and blown into cell suspension, to prepare into a certain concentration of the cell suspension. And then the osteoblasts were cultured in a cell culture plate coated with zinc ion microporous-coated titanium at the density of 1 × 104 cells/cm^2^. After culturing for 12 h, samples were fixed by glutaraldehyde and osmium, performing gradient dehydration by alcohol and replacement by isopentyl acetate. The surface was sprayed and dried at the critical point, and the adhesion and extension of the cells were observed by using scanning electron microscopy.

### 2.3. Antimicrobial Effect of Microporous Coatings Containing Zinc on *Staphylococcus aureus*

*Staphylococcus aureus-*lyophilized strain was cultured into solid culture medium, incubated in a 37°C incubator for 24 h, and transferred once for each day, to the third generation. The inoculating loop was used to scrape a little of the activated bacteria that was added into the nutrient broth medium, and the bacteria concentration of 1 × 105 cfu/ml was adjusted for application.

The ethylene oxide sterilized microporous coatings containing zinc titanium (the experimental group) and pure titanium (the control group) were placed in a petri dish. 1000 *μ*L of PBS was added to the culture dish and 100 *μ*L of the above-prepared bacteria solution was added to the surface of the two abovementioned materials, and then, incubation was performed in a constant temperature incubator at 37°C for 24 hours.

80 *μ*L of the bacterial solution was taken from the surface of the above two groups and uniformly added to the nutrient agar medium, gently applied evenly, and then placed in a 37°C incubator for 24 hours. After incubation for 24 h, the number of viable bacteria colonies on material surfaces of the above two groups was counted.

The antimicrobial ratio of the microporous coatings containing zinc material was calculated according to the following formula: *R* = (average number of colonies recovered in the blank control group – number of colonies recovered in the experimental group) / average number of colonies recovered in the blank control group × 100%.

## 3. Results

**Figure 1:**
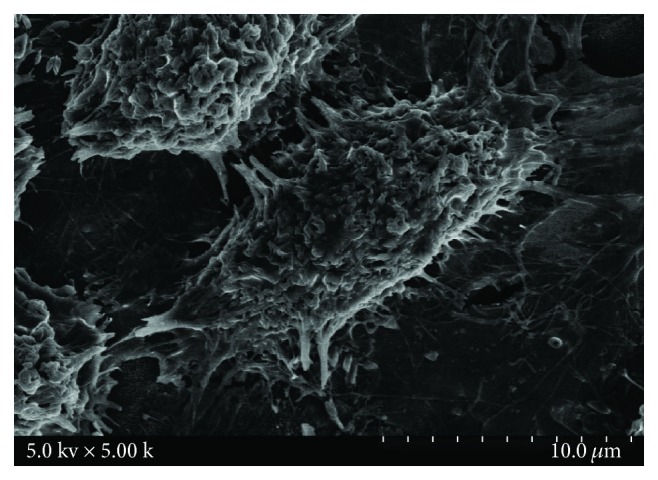
SEM of osteoblasts cultured on microporous coatings containing zinc titanium surface of 12 h cell morphology.

Figure 1 shows the morphology of osteoblasts cultured on the surface of microporous coatings containing zinc for 12 h. It clearly shows osteoblast eminentia on the surface of microporous coatings containing zinc, with large cell volume. The spreading of cells on the material surface is well, and osteoblasts tightly adhere to the surface of microporous coatings containing zinc through lamellipodia and filopodia, presenting obvious trend of the surrounding extension. The surface of cells is rough, with rich secretory granules, indicating that the osteoblasts have good adhesion and extension property on the surface of microporous coatings containing zinc.

**Figure 2:**
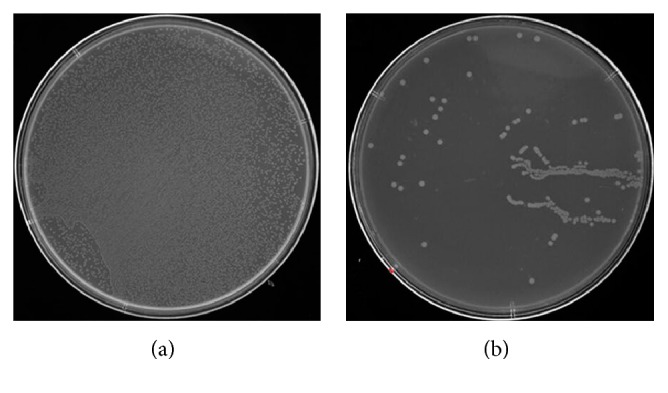
Colony count of *Staphylococcus aureus* on the sample surface after 24 h culture. (a) Pure titanium and (b) microporous coatings containing zinc titanium surface.

The antimicrobial effect of the above two materials on *Staphylococcus aureus* is shown in Figure 2, where A and B present the bacterial colony plate count photos of pure titanium surface and microporous coatings containing zinc titanium surface, respectively. We can see that in Figure 2(a), *Staphylococcus aureus* on the surface of pure titanium (control group) is densely covered with the entire culture dish.  Figure 2(b) shows the bacterial colonies on the microporous coatings containing zinc titanium surface, and the number of *Staphylococcus aureus* colony is reduced greatly. The number of colonies is very few, indicating that the microporous coatings containing zinc on titanium surface can effectively inhibit the growth of *Staphylococcus aureus* colonies, with a good antibacterial effect.

## 4. Discussion

Adhesion of cells on the surface of the material is a prerequisite and the basis for the subsequent biological behavior of cells. The quality of cell adhesion on the surface of the material determines the subsequent biological behavior of the cells, including proliferation, differentiation, and apoptosis. But the adhesion of cells on the surface of the material is an extremely complex process, and the whole process is subject to the regulation of complex signal network process [8]. It has been found that the adhesion of cells to the surface of material undergoes a number of processes such as cellular attachment, extension, and focal connection. The whole process involves the regulation of many molecular proteins, which interact with each other, inducing cell signaling and then induce transcription factors. Studies have found the physical and chemical properties of cell surfaces such as surface morphology, roughness, chemical composition, hydrophilicity, and hydrophobicity [9], and the surface charge and surface free energy interact with and promote each other; for example, for materials with proper roughness, its surface hydrophilicity and surface energy are high, and it is easy to adsorb the protein in the body fluid, and through the interaction of its secretory adhesion receptors and synthetic adhesion proteins with surface adsorbent protein, the cells will ultimately adhere to the surface of the material closely.

Studies have shown that the surface roughness of the material can promote cell adhesion, and its mechanism may be that rough surface increases the wettability and hydrophilic properties of the surface of the material, and thus promote cell adhesion. Our findings are consistent with the literature. Wu et al. [10] found that the hydrophilicity, surface roughness, and cell viability of MAO-processed material surface cells were significantly increased, which was helpful for the formation of apatite, improving the bioactivity of the titanium alloy surface, to promote the cell adhesion and proliferation. Liu et al. [11] found that the adhesion and proliferation of MAO-processed Ti-24Nb-4Zr-7.9Sn surface osteoblasts were significantly increased, with a good bioactivity and biocompatibility. Adhesion of cells was the basis of cell behavior and basis for cells to play a biological role, and it had an important impact on the subsequent cell proliferation, differentiation, mineralization, and apoptosis. In this study, we found that osteoblasts had a good adhesion in the microporous coatings containing zinc titanium surface, indicating that titanium-based metal surface microporous coatings containing zinc have a certain osteogenic performance on osteoblasts. We will further study the effect of this coating material on the proliferation, differentiation, mineralization, and apoptosis of osteoblasts in the future. And based on this, we will implement further study on the regulation of molecular mechanism of this coating material on osteoblasts to lay a good theoretical basis for clinical application.

Studies have shown that when titanium-based metals are implanted as implants, the bacteria and host cells will be competitive to reach the surface of the implants and competition results of bacteria and host cells directly affect the fate of the implants [12, 13]. If the bacteria adhere to the surface of the implants at first, it will quickly proliferate on the surface, and the result will inhibit adhesion and proliferation of osteoblasts in the implants' surface, resulting in infection. In contrast, if osteoblasts adhere to the surface of the implants at first, the bacteria will be inhibited from adhering to and proliferating on the surface. Studies have shown that the ability of anti-infection increases along with the increase of biological activity and biocompatibility of implanted materials, which may be closely related to competitive inhibition between bacteria and host cells. In view of the current view, we design to achieve excellent surface morphology, good biocompatibility, and biological activity of implants by surface modification, thereby reducing or inhibiting the occurrence of implant-related infections.

As an inorganic antibacterial agent, metal element has been widely used in clinical practice, such as the clinically used nanosilver application and sulfadiazine argentum (SD-Ag) used in the burn department, which have all achieved good clinical efficacy. Zinc, as another important inorganic antimicrobial agent, has been proved to have a good antibacterial effect, but the specific mechanism is still unknown. It is believed that many factors may act together, involving bacterial synthetase, local microenvironment of bacteria, etc.

In the present study, we found that microporous coatings containing zinc titanium have good antibacterial effect, but the exact mechanism is not known and still awaits further exploration. The existing research shows that microporous coatings containing zinc can produce a lot of reactive oxygen species, especially hydrogen peroxide, which is a strong oxidizing agent that reduces bactericidal activity, finally leading to the reduction of adhered bacteria on the coating surface. Further study reveals that when the zinc ions interact with the bacteria, zinc ions are firmly adsorbed on the cell membrane with the Coulomb force because the cell membrane is negatively charged and the zinc ion is positively charged and further penetrate the cell walls of bacteria. Subsequently, the zinc ions interact with protein, thiol (-SH), and amino (-NH2) in nucleic acid, which cause bacterial protein denaturation, destruction of bacterial synthetase activity, lower environmental pH value, etc. Under the combined effects of these factors, ultimately, it will lead to the death of bacteria due to loss of proliferation capability. Our study results are consistent with the literature reported. Jin et al. [14] prepared the coatings containing zinc on the surface of titanium alloy by ion implantation technology, and in vitro studies show that it has antibacterial and osteogenic properties, with good application prospects. Reyes-Vidal et al. [15] prepare zinc/silver composite coating by electrodeposition, and results show that the composite coating had a good antibacterial effect. Compared with these preparation methods, the microporous coatings containing zinc prepared by MAO technology in this study combined strongly with the substrate, and more importantly, zinc was uniformly doped on the surface of the coating, achieving slow release when implanted in the body, so it has antibacterial and osteogenic properties, with a good application prospect.

The integration of surface structure and biomedical function of medical metal materials is a challenging and innovative idea in the field of metal implantation. It is also a hot and difficult point in the research of surface interface of metal implant materials. Using the appropriate release of human beneficial elements to achieve the biomedical function of a medical metal material is a valuable study with important clinical application values. Through the study of effects and antimicrobial properties of microporous coatings containing zinc on the biological behavior of osteoblasts, it aims to provide a new idea and method for promoting long-term and permanent biostability of implants, with an important theoretical significance and application prospect.
